# T‐type calcium channels contribute to NMDA receptor independent synaptic plasticity in hippocampal regular‐spiking oriens‐alveus interneurons

**DOI:** 10.1113/JP273695

**Published:** 2017-03-22

**Authors:** Elizabeth Nicholson, Dimitri M. Kullmann

**Affiliations:** ^1^ University College London Institute of NeurologyLondonUK

**Keywords:** calcium channels, interneurons, synaptic plasticity

## Abstract

**Key points:**

- Regular‐spiking interneurons in the hippocampal stratum oriens exhibit a form of long‐term potentiation of excitatory transmission that is independent of NMDA receptors but requires co‐activation of Ca^2+^‐permeable AMPA receptors and group I metabotropic glutamate receptors.
- We show that T‐type Ca^2+^ channels are present in such interneurons.
- Blockade of T‐type currents prevents the induction of long‐term potentiation, and also interferes with long‐lasting potentiation induced either by postsynaptic trains of action potentials or by pairing postsynaptic hyperpolarization with activation of group I metabotropic receptors.
- Several Ca^2+^ sources thus converge on the induction of NMDA receptor independent synaptic plasticity.

**Abstract:**

NMDA receptor independent long‐term potentiation (LTP) in hippocampal stratum oriens‐alveus (O/A) interneurons requires co‐activation of postsynaptic group I metabotropic glutamate receptors (mGluRs) and Ca^2+^‐permeable AMPA receptors. The rectification properties of such AMPA receptors contribute to the preferential induction of LTP at hyperpolarized potentials. A persistent increase in excitatory transmission can also be triggered by exogenous activation of group I mGluRs at the same time as the interneuron is hyperpolarized, or by postsynaptic trains of action potentials in the absence of presynaptic stimulation. In the present study, we identify low‐threshold transient (T‐type) channels as a further source of Ca^2+^ that contributes to synaptic plasticity. T‐type Ca^2+^ currents were detected in mouse regular‐spiking O/A interneurons. Blocking T‐type currents pharmacologically prevented LTP induced by high‐frequency stimulation of glutamatergic axons, or by application of the group I mGluR agonist dihydroxyphenylglycine, paired with postsynaptic hyperpolarization. T‐type current blockade also prevented synaptic potentiation induced by postsynaptic action potential trains. Several sources of Ca^2+^ thus converge on NMDA receptor independent LTP induction in O/A interneurons.

## Key points

- Regular‐spiking interneurons in the hippocampal stratum oriens exhibit a form of long‐term potentiation of excitatory transmission that is independent of NMDA receptors but requires co‐activation of Ca^2+^‐permeable AMPA receptors and group I metabotropic glutamate receptors.
- We show that T‐type Ca^2+^ channels are present in such interneurons.
- Blockade of T‐type currents prevents the induction of long‐term potentiation, and also interferes with long‐lasting potentiation induced either by postsynaptic trains of action potentials or by pairing postsynaptic hyperpolarization with activation of group I metabotropic receptors.
- Several Ca^2+^ sources thus converge on the induction of NMDA receptor independent synaptic plasticity.

**Abbreviations**
4‐AP: 4‐aminopyridine
d‐APV: d‐aminophosphonovalerate
CP‐AMPAR: Ca^2+^‐permeable AMPA receptor
DHPG: dihydroxyphenylglycine
LTP: long‐term potentiation
mGluR: metabotropic glutamate receptor
NMDAR: NMDA receptor
O/A: oriens‐alveus
O‐LM: oriens‐lacunosum
TEA: tetraethylammonium

## Introduction

Long‐term potentiation (LTP) in hippocampal interneurons depends critically on postsynaptic Ca^2+^ signalling (Cowan *et al*. 1998; Alle *et al*. 2001; Lapointe *et al*. 2004; Galván *et al*. 2008; Nicholson & Kullmann, 2014). The dependence of LTP induction on NMDA receptors (NMDARs), however, differs according to cell type. In the stratum radiatum of the rodent CA1, a subset of interneurons exhibits a form of LTP that resembles NMDAR‐dependent LTP in principal neurons (Lamsa *et al*. 2005). However, several interneuron subtypes, predominantly in strata pyramidale and oriens, exhibit NMDAR independent LTP, which instead depends on both Ca^2+^‐permeable AMPA receptors (CP‐AMPARs) and group I metabotropic glutamate receptors (mGluRs) (Perez *et al*. 2001; Lapointe *et al*. 2004; Lamsa *et al*. 2007; Oren *et al*. 2009; Le Duigou & Kullmann, 2011; Roux *et al*. 2013). Both CP‐AMPARs and group I mGluRs are activated upon high‐frequency stimulation of presynaptic glutamatergic axons. A striking feature of NMDAR independent LTP in some interneurons is its preferential induction when presynaptic activity coincides with postsynaptic hyperpolarization. This ‘anti‐Hebbian’ feature, contrasting with ‘Hebbian’ LTP in principal cells, can be explained by polyamine‐dependent rectification of CP‐AMPARs (Lamsa *et al*. 2007; Oren *et al*. 2009; Kullmann *et al*. 2012).

An unexplained finding is that exogenous activation of group I mGluRs with the selective agonist dihydroxyphenylglycine (DHPG) can be sufficient to induce LTP in the absence of presynaptic stimulation, when paired with postsynaptic hyperpolarization (Le Duigou & Kullmann, 2011; Le Duigou *et al*. 2015). We recently reported that LTP can also be elicited when recording from regular‐spiking oriens‐alveus (O/A) interneurons in the whole‐cell configuration of the patch clamp method, with polyamines omitted from the pipette solution to relieve voltage‐dependent blockade of CP‐AMPARs (Nicholson & Kullmann, 2014). Postsynaptic action potential trains alone also led to persistent potentiation of glutamatergic transmission. This form of potentiation occluded LTP elicited by high‐frequency stimulation, implying convergence onto a common signalling cascade.

The observation that presynaptic stimulation is dispensable for LTP under two conditions (DHPG together with hyperpolarization, or action potential trains alone in whole cell recordings) prompts the search for another source of Ca^2+^. CaV3.1 T‐type channel subunits are abundant in hippocampal interneurons, especially those in the stratum oriens (Vinet & Sík, 2006; Aguado *et al*. 2016), and T‐type Ca^2+^ currents have been reported in lacunosum‐moleculare interneurons (Fraser & MacVicar, 1991). The present study shows that T‐type Ca^2+^ channels play an unexpected role in synaptic plasticity in regular‐spiking O/A interneurons.

## Methods

### Ethical approval

Postnatal day 16–23 male C57BL/6 mice were culled by cervical dislocation in accordance with the UK Animals (Scientific Procedures) Act 1986. The mice were bred in house under conditions specified in the UK Animal Welfare Act 2006 and The Welfare of Farm Animals (England) Regulations 2007 with free access to food and water.

### Acute slice preparation

Horizontal slices (300–400 μm) were prepared using standard protocols. The slicing solution contained (in mm): 92 *N*‐methyl‐d‐glucamine‐Cl, 2.5 KCl, 1.25 NaH_2_PO_4_, 2 thiourea, 5 ascorbic acid, 3 Na pyruvate, 10 MgCl_2_, 25 d‐glucose, 30 NaHCO_3_, 0.5 CaCl_2_ and 1 sucrose. The solution was continuously gassed with 95 O_2_ and 5% CO_2_. Slices were transferred to a warmed (32**°**C) solution for 10 min and then submerged in a room temperature (20–22**°**C) solution containing (in mm): 119 NaCl, 2.5 KCl, 0.5 CaCl_2_, 1.3 MgSO_4_, 1.25 NaH_2_PO_4_, 25 NaHCO_3_ and 10 glucose, and continuously bubbled with 95% O_2_ and 5% CO_2_.

### Electrophysiology

Slices were anchored in a recording chamber with a platinum and nylon fibre harp and perfused at 3 ml min^−1^ at 32°C. Cells were visualized using infrared differential interference contrast via a 20× water immersion objective on an upright microscope (BX51W1; Olympus, Tokyo, Japan). Interneurons in the stratum oriens of CA1 were patched with 4–6 MΩ resistance recording pipettes. The data reported in the present study were obtained from O/A interneurons for which the proximal dendrites were oriented parallel to stratum pyramidale.

For voltage clamp experiments, Ca^2+^ currents were isolated using an extracellular solution containing (in mm): 60 NaCl, 50 tetraethylammonium (TEA) Cl, 3 CsCl, 2.5 KCl, 2.5 CaCl_2_, 1.3 MgSO_4_, 1.25 NaH_2_PO_4_, 25 NaHCO_3_, 10 glucose, 5 4‐aminopyridine (4‐AP) and 0.001 tetrodotoxin (TTX). The pipette solution contained (in mm): 125 Cs‐gluconate, 10 Hepes, 10 Na‐phosphocreatine, 8 NaCl, 4 Mg‐ATP, 0.3 Na_3_‐GTP, 0.2 EGTA, 5 TEA‐Cl and 0.5 biocytin. Cell capacitance and series resistance were compensated (the latter by at least 60%), using a Multiclamp 700 B amplifier (Molecular Devices, Sunnyvale, CA, USA). The liquid junction potential for these recording conditions was +17 mV and all results are adjusted accordingly. Cells were held at –107 mV, and a –P/4 protocol was applied to subtract passive currents. Voltage‐dependent kinetics were fitted in OriginPro, version 9 (OriginLab Corp., Northampton, MA, USA).

For current clamp experiments, the extracellular perfusion solution contained (in mm): 119 NaCl, 2.5 KCl, 2.5 CaCl_2_, 1.3 MgSO_4_, 1.25 NaH_2_PO_4_, 25 NaHCO_3_ and 10 glucose. NMDA, GABA_A_ and GABA_B_ receptors were blocked routinely with 50 μm d‐aminophosphonovalerate (d‐APV), 100 μm picrotoxin and 1 μm CGP 52432. The pipette solution contained (in mm): 145 K‐gluconate, 8 NaCl, 20 KOH‐Hepes, 0.2 EGTA and 0.5 biocytin. No polyamines were added. Current was injected to maintain the membrane potential between –70 and –75 mV where necessary. Hyperpolarizing and depolarizing current steps were injected to elicit a ‘sag’ potential and action potentials, and interneurons that did not display a regular firing pattern typical of O/A interneurons, such as fast spiking basket cells (Sik *et al*. 1995) or burst firing axo‐axonic cells (Buhl *et al*. 1994), were excluded. For LTP experiments, concentric bipolar electrodes, connected to a constant current isolated stimulator (Digitimer, Welwyn Garden City, UK), were positioned in the alveus/stratum oriens border, 100–300 μm either side of the patched cell. The stimulus duration was 100 μs and intensity was between 20 and 320 μA, eliciting an EPSP whose baseline amplitude was in the range 2–6 mV. Synaptic plasticity was induced in one of three ways: (i) a 10 min bath‐application of 5 μm DHPG paired with hyperpolarization to –90 mV; (ii) action potential trains elicited in the postsynaptic cell with current injection (500 pA for 500 ms, repeated 20 times at 0.4 Hz) without concurrent presynaptic stimulation; and (iii) tetanic stimulation consisting of 100 pulses at 100 Hz, delivered twice with a 20 s interval delivered to one pathway, with the other used as a control.

Data were acquired using a PCI‐6221 interface (National Instruments, Austin, TX, USA) and custom software (LabVIEW; National Instruments). Currents or voltages were low‐pass filtered (4–5 kHz), digitized at 10–20 kHz, and analysed off‐line using LabVIEW and Pclamp, version 10 (Molecular Devices). Data are presented as the mean ± SEM.

### Histology

Slices were fixed in 4% paraformaldehyde for 12–15 h at 0–4**°**C, and then washed in phosphate‐buffered saline (PBS) and transferred to a PBS containing 0.3% Triton and 0.1% streptavidin‐Alexa‐488 for 3 h at room temperature. After washing, slices were mounted with Vectashield mounting medium (Vector Laboratories, Inc., Burlingame, CA, USA). Cells were visualized with an AxioImager microscope (Zeiss, Oberkochen, Germany).

### Drugs

TTA‐P2 was a gift from Merck and Co., Inc. (Kenilworth, NJ, USA). d‐APV was from Ascent Scientific (Bristol, UK). All other drugs were purchased from Tocris (St Louis, MO, USA).

### Statistical analysis

The initial EPSP slope (2–4 ms from onset) was measured in all cases to restrict attention to monosynaptic connections. For LTP experiments, we expressed the pathway‐specific potentiation as the ratio EPSP_test_/EPSP_control_, both normalized by their baseline values, to control for non‐specific drift. Unpaired *t* tests in Excel (Microsoft Corp, Redmond, WA, USA) were applied to compare the effects of drugs on the magnitude of potentiation.

## Results

### O/A interneurons express T‐type Ca^2+^ currents

We compared low‐threshold Ca^2+^ currents in pyramidal neurons and O/A interneurons in hippocampal slices prepared from postnatal day 16–23 mice. Neurons were recorded in the whole cell voltage clamp mode with a Cs^+^‐based pipette solution to improve the space clamp, and with Na^+^ and K^+^ channels blocked extracellularly with TTX, TEA and 4‐AP. GABA_A_ and NMDA receptors were blocked pharmacologically with picrotoxin (100 μm) and d‐APV (50 μm), and AMPA receptors were blocked with NBQX (10 μm). Because the intracellular Cs^+^ ions precluded measurement of firing patterns, we identified interneurons morphologically, and the commonest reconstructed cells were oriens‐lacunosum moleculare (O‐LM) interneurons.

**Figure 1:**
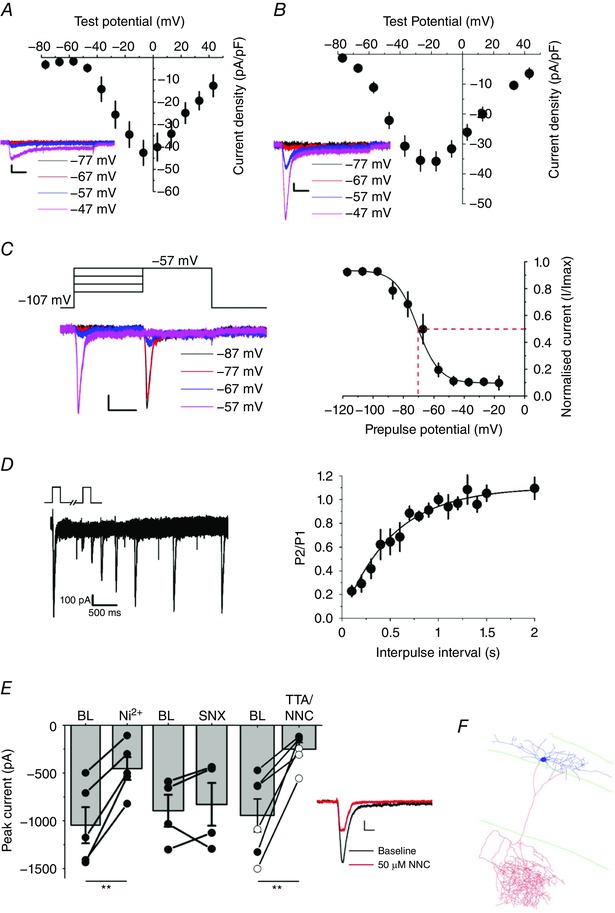
O/A interneurons express T‐type Ca^2+^ currents *A* and *B*, whole‐cell Ca^2+^ *I*–*V* relationships from pyramidal neurons (*A*, *n = *5) and O/A interneurons (*B*, *n = *24). Cells were held at –107 mV, and 600 ms depolarizing voltage commands were delivered in increments of 10 mV. Insets below: example traces. Scale bars = 100 ms, 100 pA. *C*, voltage‐dependence of inactivation. The membrane potential was stepped to –57 mV from holding potentials ranging from –107 to –17 mV (*n = *12). The normalized peak amplitudes of the currents elicited by the test pulse to –57 mV were plotted as a function of the holding current and data were fitted with a Boltzmann equation. Half‐maximal inactivation voltage was –69.9 ± 3.5 mV, as indicated by the dashed red lines (*n = *12). *D*, time‐course of recovery from inactivation. The membrane potential was held at –107 mV, and 500 ms test pulses to –57 mV were delivered with varying interpulse intervals to test the recovery of channel inactivation. The ratio of second pulse to first pulse is plotted against interpulse interval and data were fitted with a single exponential fit, yielding a time constant of 522 ms (*n = *3). *E*, left: compared to baseline (BL), peak currents were reduced by 100 μm Ni^2+^ (*n = *5, *P = *0.003), 50 μm NNC 55–0396 (filled circles, *n = *3) or 1 μm TTA‐P2 (open circles, *n = *3; pooled data *P = *0.005) but not by 100 nm SNX (*n = *4). Right: raw traces before and after 50 μm NNC 55–0396, Scale bar = 50 ms, 100 pA. Error bars indicate the SEM. *F*, example reconstruction of an OLM cell in which Ca^2+^ currents were recorded. Lines show the demarcations of strata oriens, pyramidale and the radiatum‐lacunosum moleculare border.

In CA1 pyramidal cells, depolarizing voltage steps above –39 ± 4 mV (mean ± SEM) elicited an inward current, which peaked at around –5 ± 2 mV (*n = *5) (Fig. 1 *A*), consistent with high‐threshold Ca^2+^ channels. Depolarization‐evoked currents in O/A interneurons had a *I*–*V* relationship that was shifted in a hyperpolarized direction: an inward current was detected at around –64 ± 2 mV and peaked at –22 ± 2 mV (*n = *24; comparison with pyramidal cells: *P < *0.001; repeated measures mixed ANOVA) (Fig. 1 *B*). Twenty‐seven cells were excluded from Fig. 1 *B* because of incomplete reconstructions; however, their exclusion made no substantial difference to the *I*–*V* curve. When holding the membrane potential at between –107 mV and –57 mV, the Ca^2+^ current in O/A interneurons inactivated within 100 ms, which is typical of T‐type channels (Klöckner *et al*. 1999; Hildebrand *et al*. 2009). We measured steady‐state inactivation by stepping to –57 mV after a prepulse ranging between –107 mV and –17 mV. The normalized peak amplitudes of the currents elicited by the test pulse were plotted as a function of the pre‐pulse voltage and data were fitted with a Boltzmann equation. The voltage for half‐maximal inactivation for the transient current was –69.9 ± 3.5 mV (*n = *12) (Fig. 1 *C*), which is consistent with T‐type Ca^2+^ currents (Klöckner *et al*. 1999).

Recovery from inactivation was measured by holding the membrane potential at −107 mV and delivering 500 ms test pulses to –57 mV twice with varying interpulse intervals. A monoexponential fit to the recovery of the second current amplitude gave a time constant of ∼522 ms (Fig. 1 *D*).

We tested the pharmacological profile of the transient current elicited by stepping from –107 mV to –57 mV for 600 ms, before and 5 min after application of the T‐type current blocker Ni^2+^ (100 μm). This led to a decrease in peak current to 40 ± 6% of baseline (paired *t* test, *P = *0.003) (Fig. 1 *E*). Because Ni^2+^ blocks R‐type channels in addition to T‐type channels, we tested the R‐type blocker SNX‐482 (100 μm). This failed to block the Ca^2+^ current (88 ± 12% of baseline). Two more specific T‐type Ca^2+^ channel blockers, NNC 55–0396 dihydrochloride (50 μm) and TTA‐P2 (1 μm) (Reger *et al*. 2011), also profoundly reduced the peak current (pooled data, *P = *0.005) (Fig. 1 *E*). We thus conclude that regular‐spiking O/A interneurons exhibit a prominent T‐type Ca^2+^ conductance.

### T‐type channels contribute to NMDA‐receptor independent LTP

We asked whether Ca^2+^ influx via T‐type channels contributes to synaptic plasticity. Accordingly, for the remainder of the study, we recorded from neurons in current clamp mode, using a K^+^‐based pipette solution devoid of polyamines, and evoked EPSPs by stimulating axon collaterals of local pyramidal neurons in the alveus. NMDARs were blocked throughout. We restricted attention to cells that had a sag response to hyperpolarizing current injection, and a regular firing pattern with a deep afterhyperpolarization.

**Figure 2:**
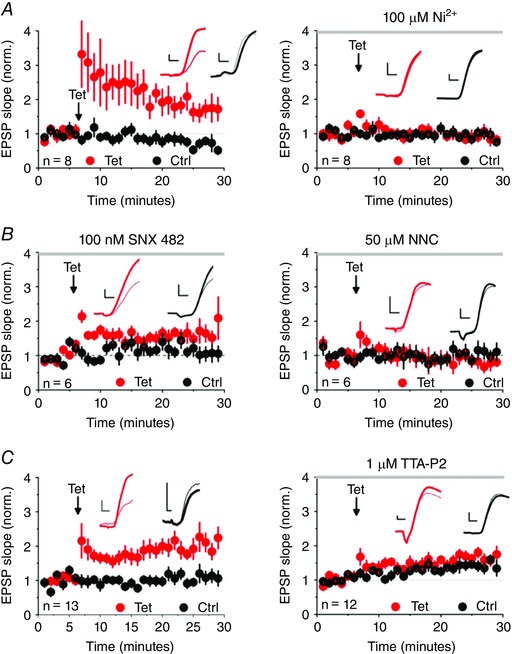
T‐type channel blockers interfere with tetanus‐induced LTP Summary plots showing pathway‐specific LTP (red, tetanized pathway, black, control pathway). *A*, LTP was prevented by Ni^2+^ (100 μm). EPSP slopes 20–25 min after tetanization, expressed as a percentage of baseline (mean ± SEM) were 271 ± 58% (interleaved controls, left) and 106 ± 19% (Ni^2+^, right; comparison: *P = *0.017, unpaired *t* test). *B*, the R‐type blocker SNX 482 (100 nm, left) failed to prevent LTP (186 ± 24%). By contrast, the T‐type blocker NNC 55–0396 (50 μm, interleaved experiments, right) abolished LTP (83 ± 18%; comparison *P = *0.01, unpaired *t* test). *C*, TTA‐P2 (1 μm, right) prevented LTP (114 ± 20%) compared to interleaved controls (177 ± 38% left, *P = *0.03, unpaired *t* test). Insets: sample averaged traces obtained 0–5 min before (pink, grey) and 20–25 min after tetanization (red, black). Scale bars = 2 ms, 1 mV.

We first examined the contribution of T‐type channels to LTP induced by presynaptic high‐frequency stimulation (2 × 100 Hz for 1 s, with a 20 s interval) paired with hyperpolarization. We systematically interleaved control experiments with experiments where blockers of Ca^2+^ channels were applied. LTP was prevented by Ni^2+^ (100 μm; comparison with interleaved controls: *P = *0.017) (Fig. 2 *A*). The R‐type blocker SNX‐482 (100 nm), in contrast, did not prevent LTP (potentiation: *P < *0.001) (Fig. 2 *B*, left), although there was a non‐significant trend towards a smaller potentiation. This argues that the effect of Ni^2+^ was a result of the blockade of T‐type and not R‐type channels. The selective blocker NNC 55–0396 (50 μm) fully prevented LTP (comparison with interleaved experiments with SNX: *P = *0.01) (Fig. 2 *B*, right). In a separate set of interleaved experiments TTA‐P2 (1 μm) also profoundly reduced LTP (comparison between control and TTA‐P2, *P = *0.037) (Fig. 2 *C*).

LTP induction is thus prevented or profoundly attenuated by three different blockers of T‐type Ca^2+^ channels.

### T‐type Ca^2+^ channels contribute to non‐associative synaptic potentiation

**Figure 3:**
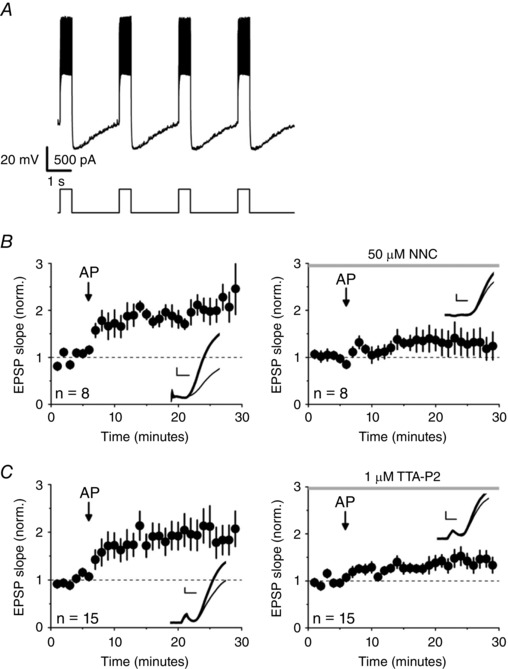
T‐type channel blockers attenuate potentiation induced by trains of postsynaptic action potentials *A*, example of action potential trains used to induce LTP; 500 ms depolarizing pulses were delivered 20 times at 0.4 Hz. *B*, Pooled data showing the effect of eliciting trains of action potentials in the absence (left; EPSP slope after 20–25 min: 198 ± 25% of baseline) and presence (right; 121 ± 27%) of 50 μm NNC 55–0396 (*P = *0.04, unpaired *t* test). *C*, TTA‐P2 (1 μm) also attenuated potentiation (left, control: 192 ± 27%; right, TTA‐P2: 132 ± 14%; *P = *0.05, unpaired *t* test). Insets: sample averaged traces obtained 0–5 min before (grey) and 15–20 min after action potential trains (black). Scale bars = 2 ms, 1 mV.

We next investigated whether T‐type calcium channels also contribute to two other forms of NMDAR independent long‐lasting potentiation of EPSPs that occur in O/A interneurons. Trains of postsynaptic action potentials alone, without presynaptic stimulation, induce a large potentiation (Nicholson & Kullmann, 2014). Control experiments (pooled data) (Fig. 3 *A*) were interleaved with experiments where the same protocol was applied in the presence of either of the specific T‐type Ca^2+^ channel blockers. Synaptic potentiation was profoundly reduced by 50 μm NNC 55–0396 (comparison with interleaved controls: *P = *0.04) (Fig. 3 *B*). A similar profound reduction in potentiation was achieved with 1 μm TTA‐P2 (*P = *0.05) (Fig. 3 *C*). The small residual potentiation may be explained by Ca^2+^ influx through high‐threshold Ca^2+^ channels or incomplete blockade of T‐type channels.

**Figure 4:**
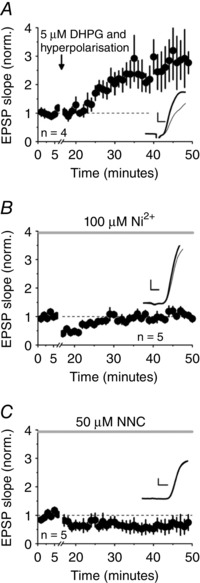
T‐type channel blockers prevent potentiation induced by DHPG and hyperpolarization *A*, pooled data showing the effect of DHPG (5 μm) paired with hyperpolarization (EPSP slope after 25–30 min: 248 ± 76% of baseline). *B*, potentiation was prevented by 100 μm Ni^2+^ (122 ± 11%; *P = *0.027, unpaired *t* test). *C*, NNC 55–0396 (50 μm) also profoundly attenuated potentiation (66 ± 30%; *P = *0.013, unpaired *t* test). Insets: sample averaged traces obtained 0–5 min before (grey) and 25–30 min after DHPG and hyperpolarization (black). Scale bars = 2 ms, 1 mV.

Synaptic potentiation can also be induced by a low concentration of the group I mGluR agonist DHPG, paired with hyperpolarizing current injection via the recording pipette (Le Duigou & Kullmann, 2011; Le Duigou *et al*. 2015). In control experiments, perfusion of DHPG (5 μm) paired with hyperpolarization for 10 min was followed by a large increase in EPSP slope (*n = *4) (Fig. 4 *A*). We interleaved this with the same protocol applied in the continuous presence of either Ni^2+^ (100 μm, *n = *5) or NNC 55–0396 (50 μm, *n = *5) to block T‐type channels. In both cases, the potentiation was prevented (comparison with controls, unpaired *t* test: *P = *0.027 and 0.013, respectively) (Fig. 4 *B* and *C*).

Both NMDAR independent LTP and non‐associative potentiation of transmission can thus be prevented or attenuated by blockade of T‐type Ca^2+^ channels.

## Discussion

The present study reveals a novel role of T‐type channels in regular‐spiking O/A interneurons in NMDAR independent synaptic potentiation, induced either using high‐frequency stimulation, or by postsynaptic action potential trains, or by pairing of postsynaptic hyperpolarization with activation of group I mGluRs. T‐type channels are thus an important source of Ca^2+^, converging with CP‐AMPARs, and interacting with group I mGluRs, in the induction of long‐lasting potentiation of excitatory synaptic transmission.

T‐type conductances are prominent in neurons that exhibit strong burst firing, such as thalamocortical (Coulter *et al*. 1989; Crunelli *et al*. 1989; Hernández‐Cruz & Pape, 1989; Suzuki & Rogawski, 1989) and reticular nucleus cells (Huguenard & Prince, 1992). Several possible mechanisms may account for the absence of strong burst firing in regular‐spiking O/A interneurons, including a high density of co‐located K^+^ channels (Golding *et al*. 1999; Wolfart & Roeper, 2002; Tsay *et al*. 2007). Indeed, T‐type currents have been isolated in many neurons that exhibit regular firing patterns, including hippocampal lacunosum‐moleculare interneurons (Fraser & MacVicar, 1991), as well as cortical somatostatin‐positive Martinotti cells that share many features with hippocampal regular‐spiking O‐LM cells (Goldberg *et al*. 2004).

Ca^2+^ influx via T‐type channels potentially explains some unusual features of synaptic plasticity in regular‐spiking O/A interneurons; in particular, the finding that presynaptic stimulation is not an obligatory requirement for potentiation of synaptic strength. This can be evoked by triggering trains of action potentials in the postsynaptic neuron (Nicholson & Kullmann, 2014), or by application of the group I mGluR agonist DHPG paired with hyperpolarization (Le Duigou & Kullmann, 2011). Group I mGluR agonists induce large inward currents in most neurons, including interneurons in stratum oriens (McBain *et al*. 1994; Woodhall *et al*. 1999), and the resulting depolarization would be expected to inactivate T‐type Ca^2+^ channels. Hyperpolarization via the recording pipette may prevent this inactivation and facilitate Ca^2+^ influx, explaining why it uncovers a long‐lasting potentiation.

Postsynaptic action potential trains or repeated depolarizing pulses have previously been shown to lead to either a transient (Kullmann *et al*. 1992) or a persistent (Kato *et al*. 2009) potentiation of glutamatergic transmission in pyramidal cells. However, L‐type rather than T‐type Ca^2+^ channels are considered to be responsible for triggering plasticity in these cells. Interestingly, non‐Hebbian LTP induced by trains of action potentials in pyramidal cells is facilitated by activation of mGluR5 receptors (Kato *et al*. 2012), implying that a convergence of signals downstream of voltage‐gated Ca^2+^ channels and group I mGluRs onto synaptic potentiation is not an exclusive property of interneurons.

A striking difference between tetanic LTP and the potentiation elicited by action potential trains or DHPG applied with hyperpolarization is that only the former exhibits pathway specificity (Lamsa *et al*. 2007; Roux *et al*. 2013). A possible explanation is that dendritic Ca^2+^ transients elicited by high‐frequency stimulation are spatially confined to the vicinity of active synapses (Topolnik *et al*. 2009).

The present study adds T‐type Ca^2+^ channels to the list of signalling cascades that have been implicated in NMDAR independent plasticity in interneurons, together with Ca^2+^‐permeable AMPA receptors (Lamsa *et al*. 2007; Kullmann & Lamsa, 2008; Oren *et al*. 2009; Roux *et al*. 2013), group I mGluRs (Perez *et al*. 2001; Lapointe *et al*. 2004), muscarinic (Le Duigou *et al*. 2015) and nicotinic receptors (Jia *et al*. 2010; Griguoli *et al*. 2013) and L‐type Ca^2+^ channels (Galván *et al*. 2008). Group I mGluRs have been shown to trigger Ca^2+^ release from intracellular stores in hippocampal interneurons and enhance Ca^2+^ influx through L‐type channels (Topolnik *et al*. 2009). Interestingly, group I mGluRs have also been reported to recruit T‐type channels in mitral cell (Johnston & Delaney, 2010) and cerebellar Purkinje cell dendrites (Hildebrand *et al*. 2009), providing a further interaction between two signalling cascades implicated in synaptic plasticity. Although the present study implies that T‐type channels are necessary for LTP induction, an important caveat is that the experiments were carried out with whole‐cell recordings with polyamines omitted from the intracellular solution. Interneuron LTP is sensitive to cytoplasmic washout and voltage‐gated Ca^2+^ channels may play a lesser role when LTP is induced with other protocols (Lamsa *et al*. 2007).

## Additional information

### Competing interests

The authors declare that they have no competing interests.

### Author contributions

Both authors designed the research. EN performed the experiments, analysed data and prepared the illustrations with the supervision of DMK. DMK wrote the paper. Both authors approved the final version of the manuscript submitted for publication. All individuals designated as authors qualify for authorship, and all those who qualify for authorship are listed.

### Funding

This research was funded by the Welcome Trust and the European Research Council.
